# Improvement of the Shock Absorption Ability of a Face Guard by Incorporating a Glass-Fiber-Reinforced Thermoplastic and Buffering Space

**DOI:** 10.1155/2018/6503568

**Published:** 2018-05-08

**Authors:** Takahiro Wada, Hiroshi Churei, Haruka Takayanagi, Naohiko Iwasaki, Toshiaki Ueno, Hidekazu Takahashi, Motohiro Uo

**Affiliations:** ^1^Department of Advanced Biomaterials, Graduate School of Medical and Dental Sciences, Tokyo Medical and Dental University, 1-5-45 Yushima, Bunkyo-ku, Tokyo 113-8549, Japan; ^2^Department of Sports Medicine and Dentistry, Graduate School of Medical and Dental Sciences, Tokyo Medical and Dental University, 1-5-45 Yushima, Bunkyo-ku, Tokyo 113-8549, Japan; ^3^Department of Oral Biomaterials Development Engineering, Graduate School of Medical and Dental Sciences, Tokyo Medical and Dental University, 1-5-45 Yushima, Bunkyo-ku, Tokyo 113-8549, Japan

## Abstract

This study aimed to evaluate the shock absorption ability of trial face guards (FGs) incorporating a glass-fiber-reinforced thermoplastic (GF) and buffering space. The mechanical properties of 3.2 mm and 1.6 mm thick commercial medical splint materials (Aquaplast, AP) and experimental GF prepared from 1.6 mm thick AP and fiberglass cloth were determined by a three-point bending test. Shock absorption tests were conducted on APs with two different thicknesses and two types of experimental materials, both with a bottom material of 1.6 mm thick AP and a buffering space of 30 mm in diameter (APS) and with either (i) 1.6 mm thick AP (AP-APS) or (ii)  1.6 mm thick GF (GF-APS) covering the APS. The GF exhibited significantly higher flexural strength (64.4 MPa) and flexural modulus (7.53 GPa) than the commercial specimens. The maximum load of GF-APS was 75% that of 3.2 mm AP, which is widely used clinically. The maximum stress of the GF-APS only could not be determined as its maximum stress is below the limits of the analysis materials used (<0.5 MPa). Incorporating a GF and buffering space would enhance the shock absorption ability; thus, the shock absorption ability increased while the total thickness and weight decreased.

## 1. Introduction

A face guard (FG) is a protector worn by an athlete that allows a speedy and safe return to play after sustaining maxillofacial traumatic injury (which can occur in contact sports [1–6]), and they are widely recognized as an effective form of treatment [7–18]. A FG must fulfill the following three requirements: (i) to protect the player from reinjury (protection ability), (ii) to avoid injury to other players (safety), and (iii) to avoid a narrowing of the player's field of vision (maintain performance) [10]. If one consults the* Laws of the Game* by the Fédération Internationale de Football Association (FIFA) [19], the first two requirements are as follows: “a player may use equipment other than the basic equipment, provided that the sole purpose is to provide physical protection and that no danger is posed to the wearer or any other players.” In addition, any effects of the FG on the field of vision must be minimized to maintain the performance of the player (requirement (iii)). Objective data taken from visual field tests have illustrated the clinical effectiveness of the FG and have demonstrated that any effects of the FG on the field of vision must be minimized [20].

A questionnaire answered by players after FG usage revealed that they were satisfied with the protective ability of the FG (requirements (i) and (ii)) but were dissatisfied with the comfort, claiming that it slipped off while playing and narrowed their field of vision; therefore, thinner and lighter FGs are required. Professional players in particular insisted on improvements to the FG's field of vision and bulkiness [15, 20].

A FG is usually constructed of a thermoplastic resin as the core material to form a certain shape and to protect the face area from damage. Its inner and outer surfaces are covered with a cushioning material. The forming temperature of the thermoplastic resin is a very important aspect of the FG fabrication process. If the required molding temperature is sufficiently low, expensive vacuum and/or pressure thermoforming machines are not required because the materials can be easily molded using hot water or hot plate in combination with applied finger pressure [11, 15, 16, 21, 22]. However, thermoplastic resins with low molding temperatures exhibit relatively low mechanical properties; therefore, FGs with these thermoplastic resins are generally thicker than those that use thermoset resins [23]. Their higher thickness leads to a decrease in the performance of the player.

To solve this problem, Abe et al. attempted to reduce the thickness of the conventional hard thermoplastic by reinforcing it with fiberglass [24, 25]. The composite material is widely known as fiber-reinforced thermoplastic (FRTP). They reported that FG constructed from FRTP have remarkable shock absorption abilities and can be manufactured to be more than 1.7 mm thinner than FG comprising conventional thermoplastics.

In clinical application, a thick hard thermoplastic material and buffering space are used to cover maxillofacial traumatic injuries to prevent any direct impact to the damaged area [15]. Takeda et al. indicated that mouth guards incorporating a hard insert and buffering space improved their shock absorption ability [26–30]. The concept consisted of using a deformation of the hard insert (similar to a structural feature termed a crumple zone or crush space used in automobiles) to absorb and diffuse the energy of the impact away from the most important area, for example, maxillofacial traumatic injured area (or tissue) and/or the most dangerous area, for example, maxillary anterior teeth.

However, the effectiveness of improving the shock absorption ability and decreasing the weight and thickness of the FG combined with a hard insert constructed from FRTP and that including a buffering space has not been verified. The purpose of this study is to evaluate the shock absorption ability of a trial FG incorporating FRTP and buffering space, and we demonstrate that the trial FG is lighter and thinner than conventional FG and has sufficient shock absorption ability.

## 2. Materials and Methods

### 2.1. Materials

Aquaplast (AP; Homecraft Rolyan, Huthwaite, North Nottingham, UK, 3.2 mm thick (AP32) and 1.6 mm thick (AP16)) was selected as the commercial thermoplastic resin for the medical splints, and a homemade glass-fiber-reinforced thermoplastic material (GF) was examined for the FG core material (Table 1).

### 2.2. Preparation of Glass-Fiber-Reinforced Thermoplastics

The homemade glass-fiber-reinforced thermoplastic materials were prepared using a previously reported method [24]. Using AP16 and sheets of plain-woven E-glass fiber cloth (M100X104H, Unitika, Osaka, Japan, with a density of 100 g·m^−2^), a vacuum hot press method was used to create GF containing four sheets of glass fiber cloth with two sheets on each outer surface [24]. The AP16 and the sheets of fiberglass cloth were separately shaped into squares with a side length of 110 mm using an ultrasonic cutter (Labo Sonic Cutter model NE87; Nakanishi Inc., Tochigi, Japan). The AP sheet was placed between the fiberglass cloth sheets and pressed using a hot press machine (modified AH-1T, AS ONE Co., Osaka, Japan) at 180°C with a final compression load of 8000 N in order to reduce the thickness to 1.5 mm. The process was carried out while evacuating using a vacuum pump (MINIVAC PD-52; Yamato Scientific Co., Tokyo, Japan).

### 2.3. Three-Point Bending Test (Specimens and Condition)

The three-point bending tests were configured according to Japanese Industrial Standards (JIS) K7171-2008 and K7074-1988 [31, 32] using a universal test machine (EZ-LX, Shimadzu Co. Ltd., Tokyo, Japan). Specimens for analysis were prepared using an ultrasonic cutter and their dimensions were measured using a micrometer (Model Number: 293-421-20; Mitsutoyo, Kanagawa, Japan, with a minimum reading of 0.001 mm). The flexural strength and modulus were calculated based on the following equations using analysis software (TRAPEZIUM X ver. 1.4.0; Shimadzu Co. Ltd., Tokyo, Japan):

1. Flexural strength = 3(*Fl*/2*bh*^2^).
2. Flexural modulus = *F*_1_*l*^3^/4*bh*^3^*d*.

*F* is the maximum load (N), *l* is the width of the support span (mm), *b* is the width (mm) of the specimen, *F*_1_ is the load (N) at any given point in the straight-line portion of the trace, and *d* is the deflection (mm) at load *F*_1_. Five specimens were examined for each material.

### 2.4. Shock Absorption Test (Specimens and Condition)

**Figure 1:**
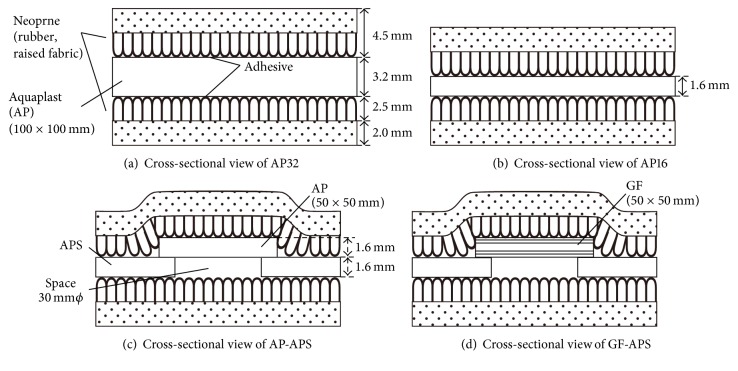
Cross-sectional views of the four investigated types of FG materials: (a) AP32 (conventional face guards), (b) AP16, (c) AP-APS, and (d) GF-APS.

A buffering space of 30 mm in diameter was incorporated into the center of the AP16 sample using an ultrasonic cutter (APS). An AP16 and GF as a cover material (a square with a side length of 50 mm) and an APS for the bottom material (a square with a side length of 100 mm) were prepared using the ultrasonic cutter. One side of the AP16, APS, and GF surface coatings was removed using dichloromethane (special grade, Sigma-Aldrich, St. Louis, MO, USA). Next, the AP16 and APS (AP-APS) and GF and APS (GF-APS) were bonded on the inside of the coating-removal surface using a hand pressed method. This is possible because the coating-removal surface has self-adhesive properties. The samples used for the shock absorption tests were covered with a cushioning material (Neoprene, Homecraft Rolyan, Huthwaite, North Nottingham, UK) on both sides using a 2 g cyanoacrylate adhesive (Aron Alpha #35045, Konishi Co., Osaka, Japan) 24 hours prior to analysis, as shown in Figure 1.

**Figure 2:**
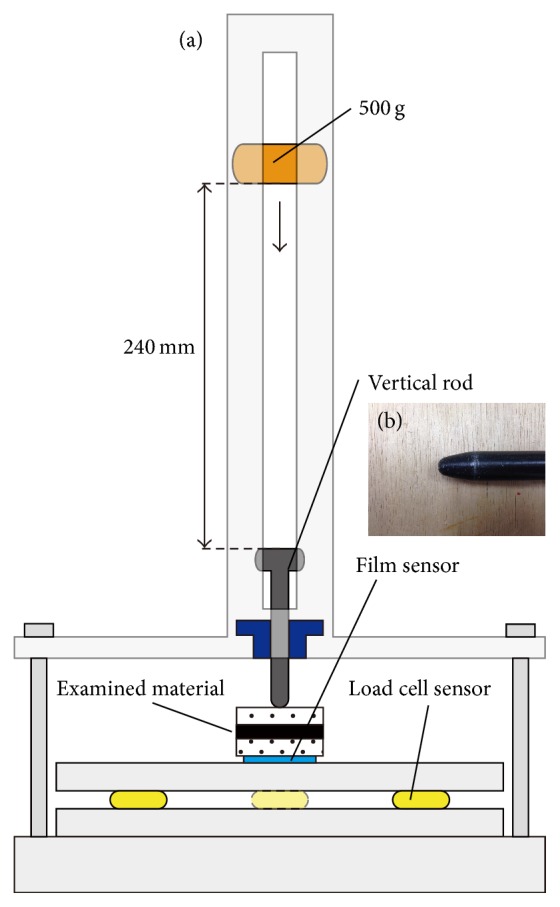
(a) Schematic diagram of the shock absorption test equipment. The experimental force of impact was applied by a free-fall drop and impact-testing machine (modified IM-201, Tester Sangyo Co., Saitama, Japan), which consisted of a free-falling object (500 g) and a vertical rod. Shock absorption performance was comparatively assessed under the two combinations of examination materials. (b) Photograph of the vertical rod.

Shock absorption tests were carried out using an impact-testing machine (modified IM-201, Tester Sangyo Co., Saitama, Japan) (Figure 2). The impact was applied to the samples by a 500 g weight dropped from a height of 240 mm onto a steel rod positioned directly above the sample with a 3/16 inch diameter rounded end. Two measuring systems were used: a load cell and a pressure-measurement film.

The impact load was measured by three dynamic compression load cells (LMB-A-2KN, Kyowa Electronic Instruments Co., Tokyo, Japan), which were placed in a triangle below a 10-mm thick stainless steel platform supporting the specimen. During the applied impact, the load was recorded by a universal recorder with data acquisition software (EDX-100A and DCS-100A, resp., Kyowa Electronic Instruments Co., Ltd., Tokyo, Japan) at a sampling rate of 20 kHz. The total impacted load could be calculated as the sum of the loads recorded by the three load cells. The maximum load after the impact was defined as the maximum load. Results taken without a specimen were recorded for reference.

The impact pressure distribution below the FG was estimated using a pressure-measurement film (Presheet, Fujifilm Corp., Tokyo, Japan), which was placed under the specimen. Films with two different sensitivities (covering pressure ranges of 2.5–1.0 MPa and 0.5–2.5 MPa, resp.) were applied. The pressed region of the film exhibits red discoloration depending on the pressure. The pressure distribution and maximum pressure were analyzed using image analysis software (Data Shot FPD-100S ver. 1.0; Fujifilm CO., Tokyo, Japan; ImageJ ver. 1.47t; National Institutes of Health (NIH), Bethesda, MD, USA [24, 25]). Five impact loads were applied to each specimen, and five specimens were examined for each set of conditions.

### 2.5. Statistical Analysis

The obtained results were analyzed using a one-way analysis of variance with Tukey's honestly significant difference test using statistical software (JMP ver. 11, SAS Institute Inc., Cary, NC, USA) with a significance level of 5%.

## 3. Results

### 3.1. Three-Point Bending Tests (Flexural Strength and Modulus)

**Figure 3:**
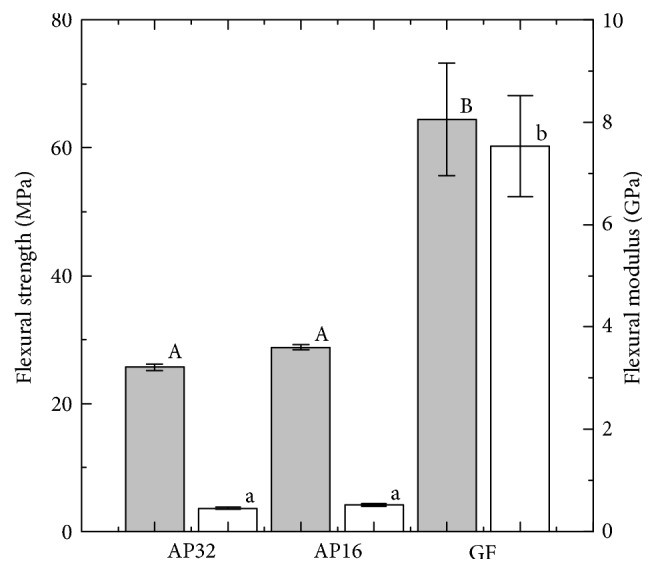
Flexural strength (left axis, gray) and modulus (right axis, white) of AP16, AP32, and GF. Bars labeled with the same letter showed no significant difference (*p* > 0.05).

Figure 3 shows the flexural strength (left axis) calculated from the three-point-bending tests. None of the specimens fractured during the experiment. The flexural strength of GF (64.4 ± 8.8 MPa) was greater than that of AP (c.a. 27 MPa (specifically, AP32: 25.7 ± 0.5 MPa, AP16: 28.8 ± 0.4 MPa)). The flexural modulus (right axis) from the three-point bending tests is also shown in Figure 3. The flexural modulus of GF (7.53 ± 0.99 GPa) was significantly greater than that of AP (c.a. 0.5 GPa (AP32: 0.46 ± 0.02 GPa, AP16: 0.52 ± 0.03 GPa)) and those of the other commercial thermoplastic resins used to prepare medical splints (0.47–2.25 GPa) [24].

### 3.2. Maximum Load during the Impact Test

**Figure 4:**
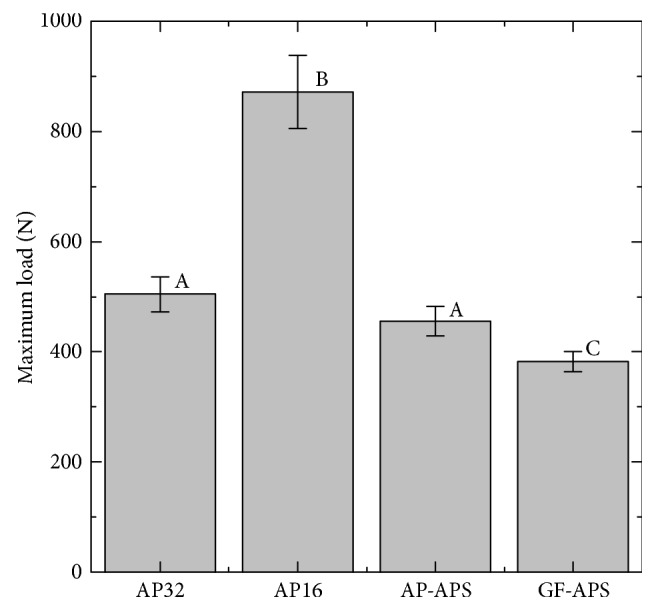
Maximum loads from the shock absorption tests. Bars labeled with the same letter showed no significant difference (*p* > 0.05).

When no specimen was included, the maximum load was 5010 ± 111 N. After inclusion, a decrease in the maximum load was observed, as shown in Figure 4. The maximum loads of AP32 and AP16 were 505 ± 32 N and 871 ± 67 N, respectively. The maximum loads of AP-APS (455 ± 27 N) and GF-APS (382 ± 18 N) were lower than those of the FG without a buffering space, that is, AP32 and AP16.

### 3.3. Pressure Distribution under the FG

**Figure 5:**
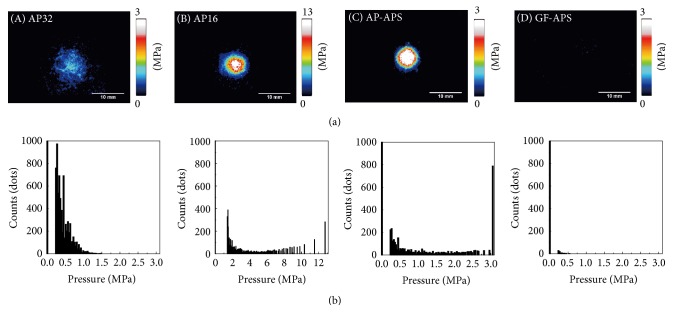
Pressure distribution (a) and pressure histogram (b) from the pressure-measurement films. (A) AP32, (B) AP16, (C) AP-APS, and (D) GF-APS. One dot represents an area of 0.125 × 0.125 mm.

**Figure 6:**
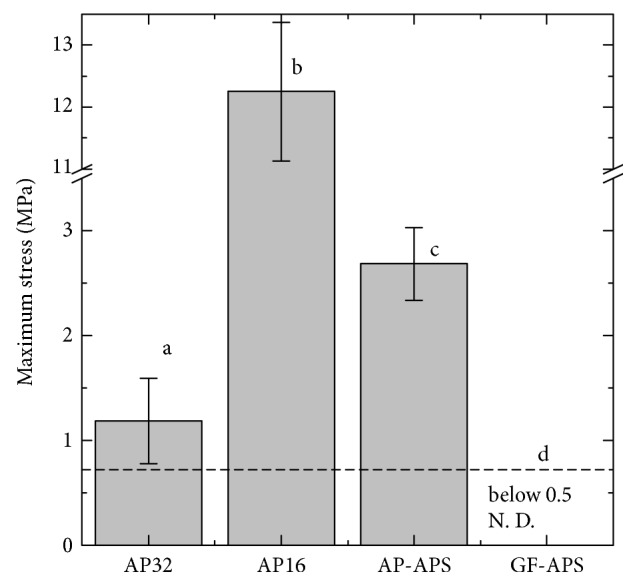
Maximum stress from the shock absorption tests. Bars labeled with the same letter showed no significant difference (*p* > 0.05).

Figure 5 shows pressure distributions and the histograms measured using the pressure-measurement film in the shock absorption tests. The maximum pressure was also analyzed from the pressure-measurement film results (Figure 6). The maximum pressure of AP16 (12.25 ± 1.12 MPa) was greater than that of the other samples. The impressed pressure areas (greater than 0.5 MPa) of AP32 and AP16 were significantly greater than that of the GF-AP samples. The maximum pressures of AP32 and AP-APS were 1.19 ± 0.41 MPa and 2.68 ± 0.52 MPa, respectively. The maximum pressure of GF-APS could not be determined because it was below the observable pressure range (0.5 MPa) of the pressure-measurement film.

## 4. Discussion

The three-point bending tests were used to determine the flexural strength and modulus. The flexural strength is determined by the maximum stress achieved during the tests, which indicates how much stress could be applied before the fracture occurred. The flexural modulus is determined by the material constant of deflection, which represents how easily the material can be bent within the elastic deformation limits. Core materials that have a higher flexural modulus covered with the cushioning material are expected to more effectively diffuse the impact, because they cause the cushioning material to compress beneath the core material [24]. GF showed greater flexural strength and modulus; therefore, it is expected to be a suitable core material.

The three load cells used in the present study can monitor the total load transmitted under the FG materials. GF-APS showed the lowest maximum load (382 ± 18 N), which was 75% of that of the conventional FG (AP32). In this study, the impact load of a free-falling weight (500 g) from a height of 240 mm (5010 ± 111 N) was used. This impact force is comparable with the impact load of fracture of the strongest part of the human maxillofacial bone (maxilla), which has been reported to be between 4930 and 5780 N [33]. The minimum load of fracture of the weakest part of the maxilla was reported to be 1088 N [34]. The results indicate that, by using GF-APS, the strong impact such as the impact of fracture of the strongest part of the maxilla is reduced to the lower impact than the impact of fracture of the weakest part of maxillofacial bone.

The impact load absorption capability, which is the ratio of the decreased impact load by the FG material compared to the original impact load, has often been discussed [33, 35, 36]. Previous research using an impact load system similar to that of the present study reported that the impact load absorption capabilities of the commercial medical splint materials ranged from 85 to 88% [35]. These results agree with the results of the present study (83 to 90%). The impact load absorption capabilities of AP-APS and GF-APS, which include a buffering space, are 91% and 92%, respectively, which indicates these specimens are superior to specimens without the buffering space (AP32 and AP16). These results suggest that the buffering space can reduce impact because of the deformation of the AP or GF.

By exhibiting a change in color to red, the pressure-measurement film can precisely record impact [37], and the level of pressure can be analyzed from the color density using a digital camera or scanner and analysis software. As previously mentioned, each pressure-measurement film has a limited range of sensitivity, and, for the present study, pressure-measurement films with two different sensitivities were employed. However, pressures below 0.5 MPa and over 10 MPa could not be detected using these films, and, as a result, pressures applied did not exceed these limits for samples analyzed (except for the reference). In general, specimens with a lower maximum pressure exhibited a smaller impressed area. FG materials with a lower maximum pressure are preferable for protecting injured areas. Therefore, the AP-APS and GF-APS are more suitable than AP16 for FGs. The maximum pressure of AP-APS is clearly higher than those of AP32 and GF-APS. The maximum pressure of GF-APS could not be detected because it was below the threshold value of the pressure-measurement films (0.5 MPa). This suggests that the degree of bending of the AP-APS cover material is larger than both the buffering space and the shock through the specimen. This is because AP is soft, and its flexural strength and modulus of AP are low. However, the degree of bending of the GF-APS cover material is smaller than that of the buffering space because the flexural strength and modulus of GF are higher than those of AP; thus, GF can sufficiently protect the buffering space, as shown in Figure 3. However, the degree of bending of the GF-APS cover material is smaller than that of the buffering space because the flexural strength and modulus of GF are higher than those of AP; thus, GF can sufficiently protect the buffering space. A decrease in the maximum pressure signifies a good dispersion of impact. These results suggest that GF-APS possesses suitable shock absorption properties for FGs, despite being relatively thin.

Including a buffering space enables constructing a FG that is thin and light and effectively absorbs shock. The thickness and weight of the core materials used in these FGs are approximately half of those that incorporate AP32 (the differences in weight between AP32 and AP16, AP-APS, and GF-APS are 16.2 g, 13.3 g and 12.9 g, resp.). The covering materials used and the thickness of the buffering space are very important to disperse and absorb impact. In this respect, AP-APS is not a suitable candidate due to its poor dispersion properties. This is because even if the material has strong absorbing properties there will still be a transmission of impact to the area of the traumatic injury. Preferably, the cover material exhibits a higher flexural modulus, for example, FRTP. Therefore, the shock absorption property of the experimental FG constructed from FRTP and including a buffering space would adequately protect injured players from impact while at the same time improving both the player's field of vision and the comfort of the FG.

The fabrication of FRTP requires a complicated process and additional costs compared to applying commercial medical splint materials without fiber such as AP. A FG requires about 250 mm × 200 mm of core materials to cover the face. Therefore, the amount of FRTP used should be minimized to supply low-cost FGs. The design adopted in this study requires small area (a square with a side length of 50 mm) of FRTP, which sufficiently covers the buffering space (30 mm in diameter). Therefore, additional costs related to the materials and the preparation time of this method are kept to a minimum. In addition, for this study, FRTP is prepared from AP; we demonstrate that because these thermoplastics have the same matrix, they can easily be joined together.

Our results are encouraging; however, this study only presents a trial FG and some tests. Future work must be carried out using the real FG in real use conditions to reveal its protective ability and other properties, such as whether it is comfortable to wear.

## 5. Conclusion

In this study, we prepared novel FG materials that incorporated GF and a buffering space. Their shock absorption ability was compared with those of commercial specimens, which increased while decreasing the total thickness and weight of the FG materials.

## Figures and Tables

**Table 1:** Materials used in the present study.

Type	Sample label	Product name	Manufacturer	Composition	Buffering space	Thickness (mm)	Weight (g)^*∗*^
Commercial medical splint	AP32	Rolyan Aquaplast-T	Homecraft Rolyan	Polycaprolactone	×	3.2	54.5 ± 0.6
	AP16				×	1.6	38.3 ± 0.4
Experimental materials	AP-APS			AP16 (50 × 50 mm)+ APS (100 × 100 mm)	○	Min 1.6–Max 3.2	41.2 ± 0.1
	GF-APS			GF (50 × 50 mm) + APS (100 × 100 mm)	○	Min 1.6–Max 3.2	41.6 ± 0.2

^*∗*^APS: AP16 with buffering space (APS);  ^*∗*^GF: glass-fiber-reinforced plastics (AP + glass fiber cloth (4 sheets)).  ^*∗*^Sample size: 100 × 100 mm, including cushioning materials.

## Abbreviations

FG: Face guard
GF: Glass-fiber-reinforced thermoplastic
AP: Aquaplast
FRTP: Fiber-reinforced thermoplastic.

## References

[1] Maladière E., Bado F., Meningaud J.-P., Guilbert F., Bertrand J.-C. (2001). Aetiology and incidence of facial fractures sustained during sports: A prospective study of 140 patients.

[2] Delilbasi C., Yamazawa M., Nomura K., Iida S., Kogo M. (2004). Maxillofacial fractures sustained during sports played with a ball.

[3] Exadaktylos A. K., Eggensperger N. M., Eggli S., Smolka K. M., Zimmermann H., Iizuka T. (2004). Sports related maxillofacial injuries: The first maxillofacial trauma database in Switzerland.

[4] Mourouzis C., Koumoura F. (2005). Sports-related maxillofacial fractures: A retrospective study of 125 patients.

[5] Fuller C. W., Junge A., Dvorak J. (2005). A six year prospective study of the incidence and causes of head and neck injuries in international football.

[6] Macisaac Z. M., Berhane H., Cray J., Zuckerbraun N. S., Losee J. E., Grunwaldt L. J. (2013). Nonfatal sport-related craniofacial fractures: Characteristics, mechanisms, and demographic data in the pediatric population.

[7] Kaplan S., Driscoll C. F., Singer M. T. (2000). Fabrication of a facial shield to prevent facial injuries during sporting events: A clinical report.

[8] Heise M., Eufinger H., Rarreck T. (2001). Individueller gesichtsschutz nach frakturversorgung am nasenbein und jochbogen bei profifußballern.

[9] Oriya S., Shiraishi M. (2001). Treatment of the professional soccer player who returned quickly to play after a mandibular fracture.

[10] Tanaka J. (2004). Sports and protectors; face guard of football player from the viewpoint of orthopedics.

[11] Morita R., Shimada K., Kawakami S. (2007). Facial protection masks after fracture treatment of the nasal bone to prevent re-injury in contact sports.

[12] Cascone P., Petrucci B., Ramieri V., TitoMatteo M. (2008). Security hi-tech individual extra-light device mask: A new protection for [soccer] players.

[13] Procacci P., Ferrari F., Bettini G., Bissolotti G., Trevisiol L., Nocini P. F. (2009). Soccer-related facial fractures: Postoperative management with facial protective shields.

[14] Ueda N., Imai Y., Ishida J. (2009). A case of faceguard fabrication for mandibular fracture of a rugby player.

[15] Churei H., Abe K., Fujino S. (2011). Clinical effectiveness of a custom faceguard for a futsal player injured with a nasal bone fracture for early and safe return: a case report.

[16] Coto N. P., Meira J. B. C., Brito e Dias R., Driemeier L., de Oliveira Roveri G., Noritomi P. Y. (2012). Assessment of nose protector for sport activities: Finite element analysis.

[17] Gandy J. R., Fossett L., Wong B. J. F. (2016). Face masks and basketball: NCAA division i consumer trends and a review of over-the-counter face masks.

[18] F.D.I. World Dent Federation (2017). FDI policy statement on Sports dentistry Adopted by the FDI General Assembly.

[19] Fédération Internationale de Football Association Laws of the game 2011/2012. http://www.fifa.com/mm/document/affederation/generic/81/42/36/lawsofthegame_2011_12e.pdf.

[20] Ueno T., Churei H., Abe K., Fujino S., Takahashi T. (2011). Clinical assessment of custom faceguards provided for sport-related maxillofacial bone fracture cases.

[21] Ueno T., Churei H. (2008). Fabrication technique for custom faceguard with thermoforming material.

[22] Fujino S., Churei H., Abe K., Miura H., Takahashi T., Ueno T. (2010). A custom faceguard for a soccer player injured with a complex fracture of the zygomatic bone and orbit: a case report (Japanese, English abstract).

[23] Abe K., Churei H., Takahashi H., Toshiaki U. (2011). Flexural properties of a faceguard core material measured by three-point bending test.

[24] Abe K., Takahashi H., Churei H., Iwasaki N., Ueno T. (2013). Flexural properties and shock-absorbing capabilities of new face guard materials reinforced with fiberglass cloth.

[25] Wada T., Churei H., Ueno T., Uo M. (2016). High shock absorbing faceguard using fiber-reinforced plastic (FRP) and elastomer.

[26] Takeda T., Ishigami K., Handa J. (2006). Does hard insertion and space improve shock absorption ability of mouthguard?.

[27] Takeda T., Ishigami K., Mishima O. (2011). Easy fabrication of a new type of mouthguard incorporating a hard insert and space and offering improved shock absorption ability.

[28] Handa J., Takeda T., Kurokawa K., Ozawa T., Nakajima K., Ishigami K. (2011). Influence of pre-laminated material on shock absorption ability in specially designed mouthguard with hard insert and space.

[29] Takeda T., Nakajima K. (2016). Improvement of impact force reduction and safety by amelioration of mouthguard design.

[30] Maeda Y., Miwa S., Gonda T. (2016). Improvement of shock absorbing capability using composite materials: Present and future.

[31] JIS K7171: 2008, Plastics - Determination of flexural property

[32] JIS K7074: 1988, The method of bending test of carbon fiber reinforecd plastics

[33] Nahum A. M., Gatts J. D., Gadd C. W., Danforth J. (1968). Impact tolerance of the skull and face.

[34] Morimoto K. (1992). Human Impact Injury Tolerance - Face.

[35] Churei H., Yokota K., Takahashi H., Ueno T. (2008). Evaluation of Fundamental Physical - properties of Face Guard Materials I - Impact Absorption.

[36] Churei H., Abe K., Yokota K., Takahashi H., Ueno T. (2010). Fundamental Evaluation of the Physical Properties of Face Guard Materials - II. Impact Absorption (Effect of Perforation Pattern).

[37] Phunthikaphadr T., Takahashi H., Arksornnukit M. (2009). Pressure transmission and distribution under impact load using artificial denture teeth made of different materials.

